# Does the correspondence between EQ-5D health state description and VAS score vary by medical condition?

**DOI:** 10.1186/1477-7525-11-155

**Published:** 2013-09-13

**Authors:** David K Whynes

**Affiliations:** 1 School of Economics, University of Nottingham, Nottingham NG7 2RD, UK

**Keywords:** EQ-5D, Visual analogue scale, Health state, Health-related quality of life, Patient-reported outcome measures

## Abstract

**Background:**

The EQ-5D health-related quality of life instrument comprises a health state classification (health problems by severity in five domains), followed by an evaluation using a visual analogue scale (VAS). Despite the EQ-5D’s use in health technology assessment and as a patient-reported outcome measure (PROM), the correspondence between the two parts of the instrument remains ill-understood. In this paper, we consider whether the association between health state classification and VAS score might vary by medical condition.

**Methods:**

EQ-5D data collected for studies of patients in four different clinical conditions or circumstances (stroke, low back pain, colposcopic investigation or cytological surveillance) were pooled to generate a sample of 3,851 patient records. VAS scores were regressed on reported problem severities, with the inclusion of intercept and slope dummy variables specific to condition.

**Results:**

The regression model achieved a goodness-of-fit of 0.54. Given its structure and the significance of the coefficients, the proportion of VAS scores which differed by condition for the same health state varied between 33.3 and 88.5 per cent of possible states.

**Conclusions:**

Many of the patients with different medical conditions or in receipt of different interventions recorded different VAS valuations, in spite of ostensibly being in the same EQ-5D-defined health states. By implication, it is probable that the same state-to-state change would by valued differently by patients experiencing different conditions.

## Introduction

The EQ-5D is a well-established and widely-used generic instrument for assessing health-related quality of life (HRQL). It is a two-part questionnaire, designed for self-completion. In the first part, the respondent describes his or her prevailing state of health in terms of perceived problem severities for five domains. These domains are limitations on mobility, capacity for self-care, ability to conduct usual activities, pain & discomfort and anxiety & depression, in that order. Three severity descriptions are available within each domain in the traditional version of the instrument (EQ-5D-3L), namely, none, moderate and severe/extreme (coded 1 through 3, respectively). This descriptive system accommodates 243 possible health states, each defined as a different vector or profile, ranging from 11111 (no problems in any domain) to 33333 (severe problems in all domains). In the second part of the instrument, the respondent evaluates his or her prevailing state of health by indicating a position on a visual analogue scale (VAS). This is a vertical, calibrated, line, anchored at 0, the “worst health state imaginable ”, and at 100, the “best health state imaginable ” [1].

The principal use of the EQ-5D instrument to date has been in health technology assessment (HTA); indeed, the UK’s influential National Institute for Health and Clinical Excellence has declared the EQ-5D to be “the preferred measure of HRQL in adults” [2] p. 38. For the purposes of cost utility HTA, the first (descriptive) part of the questionnaire is of far greater significance than the second (evaluative) part. This is because HTA conventions in most countries [3] dictate that, whilst the health effects of interventions during clinical trials should be assessed using the health state descriptions made by the participating individuals, the values of those states should derive from public or social, rather than individual, judgements [4]. These social health state utilities are obtained from independent studies which aggregate the opinions of members of general populations into state-specific “index scores”, anchored at 1 (EQ-5D state 11111) and 0 (dead).

As EQ-5D VAS results have had little relevance in HTA, the relationship between individuals’ descriptive profiles and their corresponding VAS scores has not been extensively researched. Only a few regression studies have been undertaken, predicting VAS scores from reported problem severities by domain, coded as binary dummy variables. These studies confirm the intuition that subjects who report more health problems at greater severities tend to indicate poorer HRQL in the form of lower VAS scores. For example, a UK analysis of a large sample of pooled records for surgical procedures reported that “the binary variable coefficients are all in the expected direction and are highly statistically significant. Moreover, they are consistent in each dimension, so that the coefficients on level 3 are all higher than the coefficients of level 2. The differences between the level 2 and level 3 scores are all significant” [5] p. 16-17. Such analyses also suggest that problem severity variations in the five domains are, of themselves, insufficient to explain VAS scores fully [6-8].

A new role for the EQ-5D raises further concern over the absence of investigations of the association between profiles and VAS scores. Since 2009, the National Health Service (NHS) in England has been collating and publishing data from a suite of “before and after” patient-reported outcome measures (PROMs) [9]. PROMs are intended to facilitate comparisons of provider performance and of patient benefit from services. Although the PROMs include both components of the EQ-5D, the performance of the VAS was excluded from appraisal of the pre-implementation pilot study [10]. Presently limited to four types of elective surgery (hip, knee, hernia repair and varicose veins), there exists an explicit intention to “extend PROMs across the NHS wherever practicable” [11] p. 14. Assuming that such an extension were to occur, published VAS and index scores would become available and might be used for comparison across different conditions, despite the validity of any such comparison remaining un-established. The potential problem is not confined to England, as other countries, including Sweden [12] and Canada [13], have indicated an interest in publishing EQ-5D results as part of their own PROMs packages.

The EQ-5D aims to be a “non-disease-specific instrument for describing and valuing health-related quality of life” [14] p. 337, and it was the promise of generality which made the instrument attractive as a PROM [15]. For this aspiration to be realised, however, it is necessary to suppose that the association between EQ-5D profile and VAS score does not vary systematically by medical condition or circumstances. Were the converse to be the case, it would follow that VAS results are not necessarily comparable across conditions or interventions, even when subjects are ostensibly in the same health states. In this paper, we consider the supposition as a hypothesis and investigate the association between VAS scores and health states amongst subjects experiencing different interventions.

## Method

The data for the investigation constituted a convenience sample, constructed by pooling EQ-5D records for participants in several controlled clinical trials of different conditions. The data comprised four sub-samples, as follows:

i) Men and women with a mean (SD) age of 56.5 (9.8) years, undergoing epidural steroid treatment for chronic low back pain, recorded regularly over a period of six months [16,17].

ii) Men and women with a mean (SD) age of 69.9 (11.4) years, in recovery following a stroke and recorded at approximately three months post-event [18].

iii) Women of mean (SD) age 35.2 (10.7) years, being followed up after a colposcopic examination following abnormal cervical cytology. These women would have experienced excision or ablation of abnormalities as necessary, and were recorded at approximately nine months post-event [19,20].

iv) Women of mean (SD) age 35.1 (10.7) years, with no specific medical conditions and receiving no active treatment. These women were under routine cytological surveillance following abnormal cytology, which had occurred up to one year prior to HRQL measurement [19,20].

All subjects had completed the EQ-5D-3L version of the questionnaire. Details of patients characteristics and methods of data collection appear in the sources cited. Some of the patients in the original stroke study had been recruited outside the UK but their records were omitted from this analysis, as there are grounds for believing that EQ-5D responses vary geographically [21]. The other studies had been conducted with UK subjects only.

All statistical analyses employed SPSS version 20. One-way analysis of variance was conducted to establish whether mean VAS and index scores differed between the four sub-samples. The principal hypothesis was tested using Potthoff regression analysis [22], which establishes whether a relationship between a criterion variable and predictor variables differs across values of a categorical variable. The model to be estimated is

$$Y=a+b_{1}X+b_{2}D+b_{3}D*X$$

where Y is the VAS score. X is a vector of ten predictor variables coded as 0/1 binary dummy variables. These are the presence of level 2 and of level 3 health problems for each of the five EQ-5D domains, with problems at level 1 being the reference categories. D is a vector comprising binary dummy variables to connote three of the four different sub-samples. The surveillance group was chosen as the reference category, on the grounds that patients in this group had been in receipt of the least amount of formal treatment. Finally, D*X is a vector of thirty interactions between each of the domain/severity variables and each of the conditions/interventions. Significant b_2_ or b_3_ coefficients in the regression model would contradict the primary hypothesis; specifically, one or more significant b_2_ coefficients would support the existence of different intercepts in the VAS-determining equation for those conditions, whereas significant b_3_ coefficients would support the existence of differential slopes. The regression model was fitted using stepwise regression.

## Results

A total of 3,851 records were analysed. Table 1 displays the characteristics of the four component data sets, arranged by increasing mean VAS score for each set. Analysis of variance indicated that all four mean VAS scores were significantly different from one another (F(3,3848) = 594.8, p < 0.01). The index scores of the four sets differed significantly also, with the exception of those for colposcopy and surveillance (F(3,3848) = 893.1, p < 0.01). Of the four sets, those for surveillance and colposcopy displayed the greatest similarity in terms of EQ-5D profile, with the surveillance group recording slightly fewer problems at level 1 in each domain. Compared with these two data sets, those for stroke and for back pain included substantially more problems beyond level 1 in all domains. Subjects in these two sub-samples were considerably older than those in the colposcopy and surveillance sub-samples, with the stroke patients being older on average than the back pain patients. Problems with mobility and pain & discomfort were less frequently reported in the stroke sample than in the back pain sample. In the full sample 113 different health states were represented.

**Table 1:** EQ-5D data characteristics, by data set

		**EQ-5D severity level,%**					
**Domain**	**1**	**2**	**3**	**Mean**	**95% CIs**		
**Low back pain (n = 903)**							
Mobility	5.2	93.3	1.4				
Self care	36.0	62.1	1.9				
Usual activities	6.7	81.8	11.5				
Pain & discomfort	3.3	68.7	28.0				
Anxiety & depression	42.8	46.8	10.5				
VAS score				49.3	47.8	–	50.8
Index score				0.405	0.384	–	0.426
**Stroke (n = 655)**							
Mobility	93.5	6.4	0.1				
Self care	98.1	1.9	0.0				
Usual activities	90.3	9.1	0.6				
Pain & discomfort	75.9	22.3	1.8				
Anxiety & depression	66.8	31.1	2.0				
VAS score				66.0	64.3	–	67.7
Index score				0.571	0.545	–	0.596
**Cytological surveillance (n = 1137)**							
Mobility	93.5	6.4	0.1				
Self care	98.1	1.9	0.0				
Usual activities	90.3	9.1	0.6				
Pain & discomfort	75.9	22.3	1.8				
Anxiety & depression	66.8	31.1	2.0				
VAS score				79.6	78.6	–	80.6
Index score				0.885	0.874	–	0.896
**Colposcopy (n = 1156)**							
Mobility	95.1	4.8	0.2				
Self care	99.0	1.0	0.0				
Usual activities	91.4	8.1	0.5				
Pain & discomfort	78.7	20.0	1.2				
Anxiety & depression	70.0	27.7	2.3				
VAS score				81.7	80.8	–	82.6
Index score				0.896	0.886	–	0.906

Table 2 presents the regression model (adjusted R^2^ = 0.54) which possessed a number of characteristics of interest. First, neither of the coefficients for the level 2 and the level 3 variable in the “capacity for self care” domain achieved statistical significance. Second, and irrespective of intervention, the relative magnitudes of the estimated coefficients of levels 2 and 3 for the remaining domains were consistent with intuition, in that greater problem severity was associated with lower VAS score. Third, none of the coefficients on the intercept dummies (b_2_) achieved statistical significance at 5 per cent. Fourth, as regards the slope dummies (b_3_), moderate limitations on usual activities for women following colposcopy exerted a greater negative effect on VAS score, compared with the reference group (surveillance). For those with low back pain compared with the surveillance group, pain & discomfort at levels 2 and 3 produced significantly lower VAS scores, whereas anxiety & depression at these levels were associated with higher VAS scores. For stroke patients in comparison with the reference group, both severe pain & discomfort and severe anxiety & depression were associated with smaller negative effects on VAS scores.

**Table 2:** Regression results

	**EQ-5D domain**	**Severity level**	**b**	**SE**	**p =**
	Constant		87.1	0.4	< 0.01
	Mobility	2	−4.7	1.1	< 0.01
	Mobility	3	−10.1	2.5	< 0.01
	Usual activities	2	−11.5	1.0	< 0.01
	Usual activities	3	−17.2	1.4	< 0.01
	Pain & discomfort	2	−3.9	0.8	< 0.01
	Pain & discomfort	3	−18.5	2.9	< 0.01
	Anxiety & depression	2	−11.8	0.7	< 0.01
	Anxiety & depression	3	−30.9	2.4	< 0.01
Interaction terms (D*X)					
Colposcopy	Usual activities	2	5.5	1.8	< 0.01
Back pain	Pain & discomfort	2	−11.7	1.3	< 0.01
Back pain	Pain & discomfort	3	−11.4	3.2	< 0.01
Back pain	Anxiety & depression	2	7.3	1.3	< 0.01
Back pain	Anxiety & depression	3	22.2	3.1	< 0.01
Stroke	Pain & discomfort	3	9.1	4.3	0.03
Stroke	Anxiety & depression	3	14.2	3.8	< 0.01

Insignificant coefficients for the intercept dummies (b_2_) imply that the predicted mean VAS score at health state 11111 is the same for all four conditions, namely, the constant term. However, significant coefficients on at least some of the slope dummies mean that, for many other health states as described by the EQ-5D, the predicted VAS score must vary by condition. Evaluating the estimated regression equation for vector 22222 produces mean VAS scores of 50.8 for low back pain, 55.2 for surveillance and for stroke, and 60.7 for colposcopy. The predicted scores for vector 33333 are 10.5 for surveillance and colposcopy, 21.3 for back pain, and 33.7 for stroke.

Of the 243 EQ-5D states which any respondent could possibly occupy, 81 (33.3 per cent) would have a severity level of 2 for “usual activities”. Given the model’s structure, therefore, the VAS scores for the colposcopy group would differ from those of the reference group for these 81 states. By the same token, the scores for the stroke and low back pain groups would differ for 135 and 215 states (55.6 and 88.5 per cent, respectively), compared against the reference. The scores for the stroke and back pain groups would, between themselves, also differ for 215 (88.5 per cent) of states.

The estimated regression model indicates that the marginal impact of a change in health state on VAS score would, for many such changes, differ by condition. By way of example, and based on the calculation above, a change from health state vector 33333 to vector 22222 would improve VAS scores by 21.5, 29.5, 44.7 and 50.2 for stroke, back pain, surveillance and colposcopy patients, respectively. At the domain level, and according to the coefficients presented in Table 2, a decrease in severity from level 2 to level 1 in the pain & discomfort domain would entail an increase of 3.9 in VAS score for the surveillance group. For the low back pain group, however, the improvement would register an additional increase of 11.7, or 15.6 in total. Similarly, a pain & discomfort severity reduction from level 3 to level 2 would result in the mean VAS score being 14.6 higher amongst the colposcopy group but only 5.5 higher for the stroke group. If anxiety & depression fell from severity level 3 to level 2, the mean VAS score would rise by 19.0 according to the colposcopy patients but by 4.9 according to the stroke patients. An improvement from level 2 to level 1 would imply an increase of 4.5 in VAS score for the low back pain group but of 11.8 for the surveillance group.

## Discussion

A literature on the association between EQ-5D VAS scores and health state descriptions does exist, although in a context quite different from that of the present investigation. In the past, the VAS has been employed to establish index scores, rather than to self-evaluate HRQL *per se*. Respondents from general populations have been asked to consider hypothetical health states and to associate a VAS score with each [23]. The average valuations of hypothetical states made by large numbers of individuals currently in no particular actual state have become, in effect, social health state utilities. It was observed at an early stage of the EQ-5D’s development that when respondents actually existed in the health state which they had been asked to value, their valuations differed significantly from those for whom the state was hypothetical [24,25]. The observed discrepancies between patient and public values stimulated debate over both whose opinions were the most appropriate for HTA purposes [26] and the validity of using the VAS to elicit social preferences [27].

In spite of the difference in context, two of the social valuation studies provide some support for our findings. First, the magnitude of the divergence between patient VAS valuations of real states and population VAS values for those states considered hypothetically, has been shown to vary by medical condition [28]. This particular result also contradicts the hypothesis under investigation, namely, that patient VAS scores for any particular EQ-5D health profile are not condition-specific. Second, a study investigating the marginal impact of problem severity by domain concluded that, compared to respondents who were valuing hypothetical states, those valuing the states which they were actually experiencing attributed a far lower significance to the capacity for self-care [29]. Our subjects who were valuing real states also found the capacity for self-care domain insignificant (Table 2).

The principal limitation of this study lies in its data. These were derived from trial-based economic evaluations in which the author participated and which were readily available. Convenience rather than premeditated design influenced the selection of records and, scientifically, the data are less-than-perfect as a basis for testing the hypothesis. For example, records of a proportion of the stroke patients were completed by proxies, and the back pain sub-sample comprised repeated measures whilst the remainder did not. Sequential evaluation of health states is prone to individual subject bias although none was identified in the original studies, and VAS and index scores were correlated over time [17]. That having been said, inconsistencies in repeated VAS measures have been identified; for example, a foot surgery study reported a significant improvement of 0.2 in EQ-5D index scores at six months whilst detecting no change in VAS scores [30].

Women were more heavily represented in the sample than men, with two sets including data for women only. We cannot exclude the possibility, therefore, that the observed effect should be ascribed to sex rather than to the medical conditions themselves. Opposing this possibility, (i) none of the previous studies relating VAS response to health profile reported a sex effect, (ii) regression models using both the low back pain data and the stroke data separately failed to produce significant coefficients for the sex of respondent. In similar vein, the considerable age differences between sub-samples raises the possibility that differential response in VAS scoring might be age- rather than condition-related. Distinguishing the effect would prove difficult in this sample, in view of the strong association between sub-sample age and condition.

In view of the new importance attached to VAS scores, further research would seem imperative. Ideally, the hypothesis would be investigated using purposefully-collected data including more variables. Two classes of candidate for inclusion would be, first, health domains which are absent from the EQ-5D but present in other HRQL instruments, domains such as sleep, memory and energy & fatigue [8] and, second, measures of psychological disposition which have been shown to influence self-perceived health independently of actual health [31,32]. Even so, and in spite of the idiosyncrasies of the data, the coefficient of determination for the Table 2 model was considerably beyond the 0.24-0.32 range obtained in previous regression models [5-7] where different medical conditions had not been distinguished.

## Conclusions

The results call into question the proposition that patients in the same EQ-5D-defined health state but with different medical conditions (or in receipt of different interventions) necessarily evaluate the state similarly. The regression model supports the possibility that a given change between two EQ-5D-defined health states could produce different changes in VAS scores for different medical conditions and interventions. It follows that EQ-5D VAS scores published as PROMS may not be consistent across different conditions or interventions.

## Abbreviations

HRQL: Health-related quality of life; HTA: Health technology assessment; NHS: National health service; PROM: Patient-reported outcome measure; VAS: Visual analogue scale.

## Competing interests

The author declares that he has no competing interests.

## References

[B1] Rabin R Oemar M Oppe M on behalf of the EuroQoL Group EQ-5D-3L user guide 2011 4 Rotterdam: EuroQoL Group

[B2] National Institute for Health and Clinical Excellence Guide to the methods of technology appraisal 2008 London: NICE 27905712

[B3] Sorenson C Drummond M Kanavos P Ensuring value for money in health care: the role of health technology assessment in the European Union 2008 Copenhagen: World Health Organisation

[B4] Benzer M Independence in dependence: health technology assessment, quality of life, and the position of the patient 2013 London: Centre for Analysis of Risk and Regulation, London School of Economics and Political Science

[B5] Feng Y Parkin D Devlin NJ Assessing the performance of the EQ-VAS in the NHS PROMs programme (Research Paper 12/01) 2012 London: Office of Health Economics 10.1007/s11136-013-0537-z PMC4287662 24081873

[B6] Jelsma J Ferguson G The determinants of self-reported health-related quality of life in a culturally and socially diverse South African community Bull World Health Organ 2004 82 206 212 15112009 PMC2585936

[B7] Whynes DK Correspondence between EQ-5D health state classifications and EQ VAS scores Health Qual Life Outcomes 2008 6 94 10.1186/1477-7525-6-94 18992139 PMC2588564

[B8] Perneger TV Courvoisier DS Exploration of health dimensions to be included in multi-attribute health-utility assessment Int J Qual Health Care 2011 23 52 59 10.1093/intqhc/mzq068 21084324

[B9] Health & Social Care Information Centre Provisional monthly patient reported outcome measures (PROMs) in England: a guide to PROMs methodology 2013 5 Leeds: H&SCIC

[B10] Browne J Jamieson L Lawsey J van der Meulen J Black N Cairns J Lamping D Smith S Copley L Horrockes J Patient Reported Outcome Measures (PROMs) in elective surgery: report to the Department of Health 2007 London: Health Services Research Unit, London School of Hygiene & Tropical Medicine

[B11] Department of Health Equity and excellence: liberating the NHS (Cm 7881) 2010 Norwich: The Stationery Office

[B12] Rolfson O Kärrholm J Dahlberg LE Garellick G Patient-reported outcomes in the Swedish hip arthroplasty register J Bone Joint Surg 2011 93-B 867 875 10.1302/0301-620X.93B7.25737 21705555

[B13] Bryan S Broesch J Dalzell K Davis J Dawes M Doyle-Waters MM Lewis S McGrail K McGregor MJ Murphy JM Sawatzky R What are the most effective ways to measure patient health outcomes of primary health care integration through PROM (Patient Reported Outcome Measurement) instruments? 2013 Vancouver, BC: Centre for Clinical Epidemiology & Evaluation

[B14] Rabin R De Charro F EQ-5D: a measure of health status from the EuroQol group Ann Med 2001 33 337 343 10.3109/07853890109002087 11491192

[B15] Devlin NJ Parkin D Browne J Patient-reported outcome measures in the NHS: new methods for analysing and reporting EQ-5D data Health Econ 2010 19 886 905 10.1002/hec.1608 20623685

[B16] Whynes DK McCahon RA Ravenscroft A Hardman J Cost effectiveness of epidural steroid injections to manage chronic lower back pain BMC Anesthesiol 2012 12 26 10.1186/1471-2253-12-26 23016755 PMC3468401

[B17] Whynes DK McCahon RA Ravenscroft A Hodgkinson V Evley R Hardman JG Responsiveness of the EQ-5D health-related quality-of-life instrument in assessing low back pain Value Health 2013 16 124 132 10.1016/j.jval.2012.09.003 23337223

[B18] Whynes DK Sprigg N Selby J Berge E Bath PM for the ENOS Investigators Testing for differential item functioning within the EQ-5D Med Decis Making 2013 33 252 260 10.1177/0272989X12465016 23184462

[B19] TOMBOLA Group Cytological surveillance compared with immediate referral for colposcopy in management of women with low grade cervical abnormalities: multicentre randomised controlled trial Br Med J 2009 339 b2546 19638646 10.1136/bmj.b2546 PMC2718083

[B20] TOMBOLA Group Options for managing low grade cervical abnormalities detected at screening: cost effectiveness study Br Med J 2009 339 b2549 19638648 10.1136/bmj.b2549 PMC2718086

[B21] Bailey H Kind P Preliminary findings of an investigation into the relationship between national culture and EQ-5D value sets Qual Life Res 2010 19 1145 1154 10.1007/s11136-010-9678-5 20496167

[B22] Potthoff RF Statistical aspects of the problem of biases in psychological tests (Institute of Statistics Mimeo Series No 479) 1966 Chapel Hill, NC: Department of Statistics, University of North Carolina

[B23] Greiner W Weijnen T Nieuwenhuizen M Oppe S Badia X Busschbach J Buxton M Dolan P Kind P Krabbe P A single European currency for EQ-5D health states. results from a six-country study Eur J Health Econ 2003 4 222 231 10.1007/s10198-003-0182-5 15609189

[B24] Dolan P The effect of experience of illness on health state valuations J Clin Epidemiol 1996 49 551 564 10.1016/0895-4356(95)00532-3 8636729

[B25] McPherson K Myers J Taylor WJ McNaughton HK Weatherall M Self-valuation and societal valuations of health state differ with disease severity in chronic and disabling conditions Med Care 2004 42 1143 1151 10.1097/00005650-200411000-00014 15586842

[B26] Gandjour A Theoretical foundation of patient v. population preferences in calculating QALYs Med Decis Making 2010 30 E57 E63 10.1177/0272989X10370488 20511562

[B27] Parkin D Devlin N Is there a case for using visual analogue scale valuations in cost-utility analysis? Health Econ 2006 15 653 664 10.1002/hec.1086 16498700

[B28] Mann R Brazier JE Tsuchiya A A comparison of patient and general population weightings of EQ-5D dimensions Health Econ 2009 18 363 372 10.1002/hec.1362 18702081

[B29] Rand-Hendriksen K Augestad LA Kristiansen IS Stavem K Comparison of hypothetical and experienced EQ-5D valuations: relative weights of the five dimensions Qual Life Res 2012 21 1005 1012 10.1007/s11136-011-0016-3 21932138

[B30] Maher AJ Kilmartin TE An analysis of Euroqol EQ-5D and Manchester Oxford foot questionnaire scores six months following podiatric surgery J Foot Ankle Res 2012 5 17 10.1186/1757-1146-5-17 22776703 PMC3489846

[B31] Goodwin R Engstrom G Personality and the perception of health in the general population Psychol Med 2002 32 325 332 10.1017/S0033291701005104 11866326

[B32] Kostka T Jachimowicz V Relationship of quality of life to dispositional optimism, health locus of control and self-efficacy in older subjects living in different environments Qual Life Res 2010 19 351 361 10.1007/s11136-010-9601-0 20146007

